# Distinct Properties of Human M-CSF and GM-CSF Monocyte-Derived Macrophages to Simulate Pathological Lung Conditions In Vitro: Application to Systemic and Inflammatory Disorders with Pulmonary Involvement

**DOI:** 10.3390/ijms19030894

**Published:** 2018-03-17

**Authors:** Alain Lescoat, Alice Ballerie, Yu Augagneur, Claudie Morzadec, Laurent Vernhet, Olivier Fardel, Patrick Jégo, Stéphane Jouneau, Valérie Lecureur

**Affiliations:** 1 Univ Rennes, CHU Rennes, Inserm, EHESP, Irset (Institut de recherche en santé, environnement et travail)-UMR_S 1085, F-35000 Rennes, France; Alain.lescoat@chu-rennes.fr (A.L.); alice.ballerie@chu-rennes.fr (A.B.); yu.augagneur@univ-rennes1.fr (Y.A.); claudie.morzadec@univ-rennes1.fr (C.M.); laurent.vernhet@univ-rennes1.fr (L.V.); olivier.fardel@univ-rennes1.fr (O.F.); patrick.jego@chu-rennes.fr (P.J.); stephane.jouneau@chu-rennes.fr (S.J.).; 2 Department of Internal Medicine, Rennes University Hospital, 35203 Rennes, France; 3 Pôle Biologie, Rennes University Hospital, 35203 Rennes, France; 4 Department of Respiratory Diseases, Rennes University Hospital, 35203 Rennes, France

**Keywords:** macrophages, polarization, M-CSF, GM-CSF, lung, interstitial lung disease, lung cancers, systemic sclerosis, sarcoidosis, flow cytometry

## Abstract

Macrophages play a central role in the pathogenesis of inflammatory and fibrotic lung diseases. However, alveolar macrophages (AM) are poorly available in humans to perform in vitro studies due to a limited access to broncho-alveolar lavage (BAL). In this study, to identify the best alternative in vitro model for human AM, we compared the phenotype of AM obtained from BAL of patients suffering from three lung diseases (lung cancers, sarcoidosis and Systemic Sclerosis (SSc)-associated interstitial lung disease) to human blood monocyte-derived macrophages (MDMs) differentiated with M-CSF or GM-CSF. The expression of eight membrane markers was evaluated by flow cytometry. Globally, AM phenotype was closer to GM-CSF MDMs. However, the expression levels of CD163, CD169, CD204, CD64 and CD36 were significantly higher in SSc-ILD than in lung cancers. Considering the expression of CD204 and CD36, the phenotype of SSc-AM was closer to MDMs, from healthy donors or SSc patients, differentiated by M-CSF rather than GM-CSF. The comparative secretion of IL-6 by SSc-MDMs and SSc-AM is concordant with these phenotypic considerations. Altogether, these results support the M-CSF MDM model as a relevant in vitro alternative to simulate AM in fibrotic disorders such as SSc.

## 1. Introduction

Macrophages constitute a heterogeneous population of mononuclear cells. They can indeed adopt various degrees of polarization with distinct functional and phenotypic properties in vivo and in vitro [1]. Depending on these polarization characteristics, macrophages can be clustered, on one hand, into pro-inflammatory macrophages, called M1 macrophages or classically activated macrophages [2], and, on the other hand, into alternatively activated macrophages or M2 macrophages, involved in the resolution of inflammation and participating in tissue remodeling [3]. These M1/M2 states of polarization constitute the extreme borders of a wide continuous functional and phenotypic spectrum, reflecting the plasticity and heterogeneity of human macrophages [4,5]. A better understanding of macrophage polarization and of the cytokines and growth factors shaping this heterogeneity is also especially relevant to explore the pathogenesis of inflammatory lung diseases [6,7,8]. Indeed, an imbalanced polarization of lung macrophages has been shown to be involved in the pathogenesis of inflammatory interstitial lung diseases (ILD) and systemic conditions with pulmonary involvement [7]. An excessive M2 polarization has been described in the pathogenesis of some inflammatory systemic autoimmune disorders such as Systemic sclerosis (SSc) [7,9]. SSc is a chronic autoimmune disease characterized by an early inflammation of small vessels followed by fibrotic manifestations such as skin fibrosis and interstitial lung disease with pulmonary fibrosis [10]; however, the pathogenesis of SSc-associated ILD is still poorly understood. Beyond fibrotic disorders, alveolar macrophages (AM) are also involved in the pathogenesis of granulomatous diseases such as sarcoidosis and neoplastic disorders, like lung cancers [1].

Pulmonary macrophages are poorly available in human to perform in vitro studies due to a limited access to broncho-alveolar lavage (BAL) and to an even more limited access to interstitial macrophages [7]. The current model to mimic pulmonary macrophages in vitro is blood monocyte-derived macrophages (MDMs). Two MDM lineages exist in vitro depending on the growth factor used for their differentiation: GM-CSF MDMs or M-CSF MDMs [11]. GM-CSF and M-CSF are directly involved in macrophage polarization: GM-CSF is indeed classically associated with the M1 polarization process whereas M-CSF participates in M2 polarization [12]. Nonetheless, recent publications have demonstrated that our view on macrophage polarization and the involvement of M-CSF and GM-CSF in this complex process has become too bipolar [13]. M-CSF MDMs for example, can also exert M1 pro-inflammatory properties [11]. In vivo, M-CSF and GM-CSF are also both necessary for a balance lung physiology. On one hand, M-CSF deficient mice show a lack of tissue macrophages and an ineffective response against mycobacterium tuberculosis lung infection, illustrating a role of this growth factor for a proper macrophage function [14]. On the other hand, patients suffering from an auto-immune driven deficiency in GM-CSF, develop a severe lung condition called pulmonary alveolar proteinosis, characterized by an accumulation of surfactant and cell debris in the alveolar lumen due to an ineffective macrophage maturation, demonstrating the major role of GM-CSF in AM physiology [15]. GM-CSF MDMs have therefore been considered for a long time as the most relevant model to simulate pulmonary macrophages in vitro. However, the expanding knowledge on macrophage polarization in human and the recent more refined phenotypic characterization of M1 and M2 macrophages in inflammatory and fibrotic lung disorders, call for a revaluation of this statement [13].

This study aims: a/to further characterize the phenotypic properties of GM-CSF/M-CSF MDMs and b/to confront these characteristics to those of AM obtained from BAL of patients suffering from various inflammatory and/or fibrotic disorders with pulmonary involvement, i.e., lung cancers, sarcoidosis and SSc.

## 2. Results

### 2.1. Phenotype of M-MDMs, GM-MDMs and AM

The phenotype of all AM, regardless of their origin (*n* = 16 patients, with lung neoplasia (*n* = 6), sarcoidosis (*n* = 5) and SSc-ILD (*n* = 5)), plated for 24 h after BAL fluid recovery, was compared to MDMs from healthy blood donors, differentiated in the presence of GM-CSF (GM-MDMs) or M-CSF (M-MDMs) for 6 days. Two panels of four membrane markers were used to characterize these cells. Flow cytometry graphs are shown in supplementary Figure S1.

**Figure 1:**
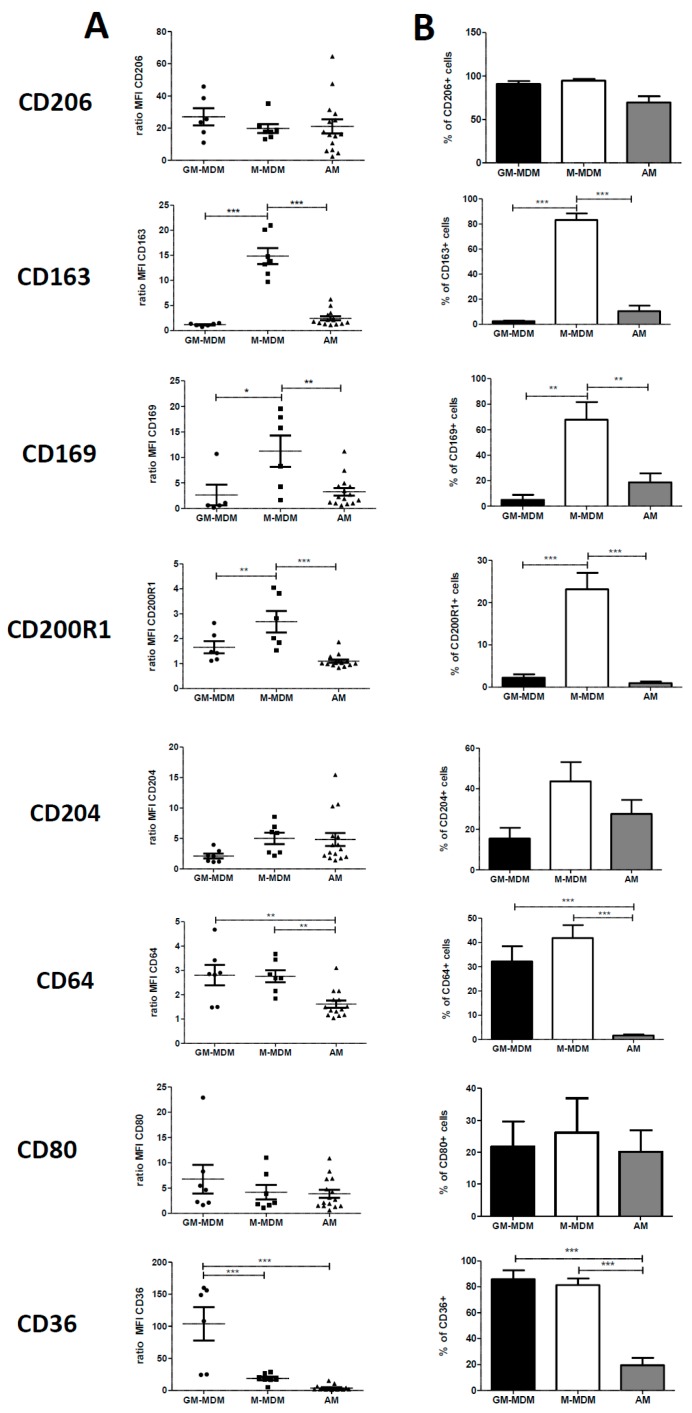
Comparison of cell surface molecule expression of alveolar MΦ (AM) and GM-CSF or M-CSF-derived MDMs (monocyte-derived macrophages). Primary human monocytes from healthy donors were differentiated into MDMs in vitro in the presence of GM-CSF (GM-MDMs) or M-CSF (M-MDMs) for 6 days. Bronchoalveolar lavage fluids of patients were washed and cells were plated until the following day. Cells were then harvested, stained and the expression of cell surface molecules was analyzed by flow cytometry. Data are expressed as mean fluorescence intensity (MFI) relative to isotype control (ratio) +SEM (**A**) and as percentage of positive cells + SEM (**B**) for at least six healthy donors and 14 or 15 AM. ANOVA followed by Newman–Keuls’ multiple comparison Test, * *p* < 0.05, ** *p* < 0.01 and *** *p* < 0.001.

CD206, a transmembrane protein also called mannose receptor, was highly expressed in the three types of macrophages without any difference in its expression and in the percentage of CD206+ cells. (Figure 1). The expressions of CD163 (or haemoglobin/haptoglobin receptor), CD169 (or Siglec 1) and CD200R1 were significantly higher in M-MDMs than GM-MDMs or than AM (Figure 1). The expression of CD64 (or FcγR1) was comparable between GM-MDMs and M-MDMs cells whereas it was significantly decreased in AM. Concerning the scavenger receptor CD36, we observed a lower expression of this molecule in M-MDMs and AM in comparison with GM-MDMs and a lower percentage of positive cells in AM in comparison with both M-MDMs and GM-MDMs (Figure 1). Finally, the expressions of the scavenger receptor A also called CD204 and the costimulatory marker CD80 were similar between the three types of macrophages, with a ratio of MFI below 10 in all groups (Figure 1). Altogether, we found that four membrane markers (CD163, CD169, CD200R1 and CD36), among the eight studied, were differentially expressed between GM-MDMs and M-MDMs. Therefore, our data illustrated that the phenotype of AM taken as a whole, without considering each lung disease separately, was closer to GM-MDM (similar expression of CD163, CD169, CD200R1) than to M-MDM phenotype.

### 2.2. Phenotypic Differences among AM of Patients Suffering from Lung Neoplasia, Sarcoidosis and SSc Associated ILD

**Figure 2:**
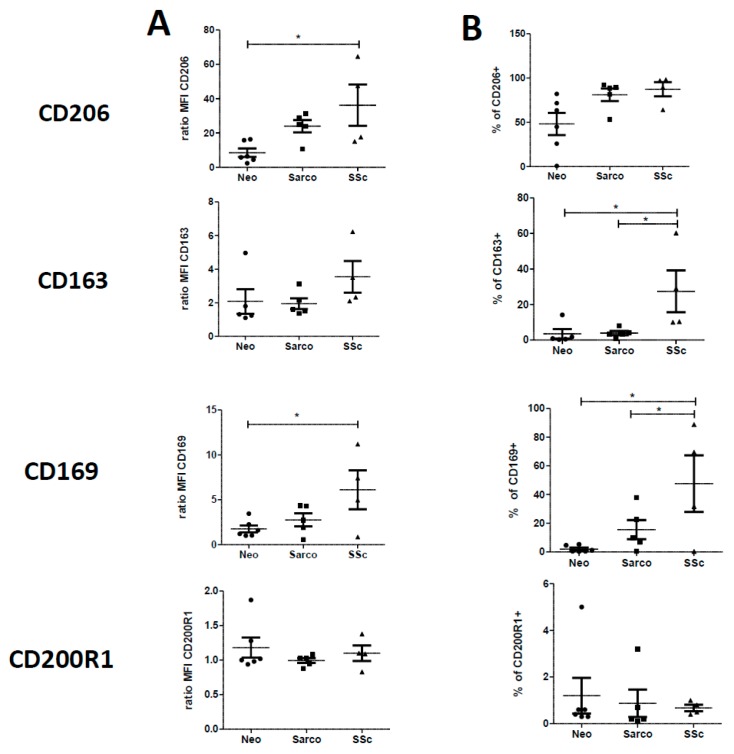

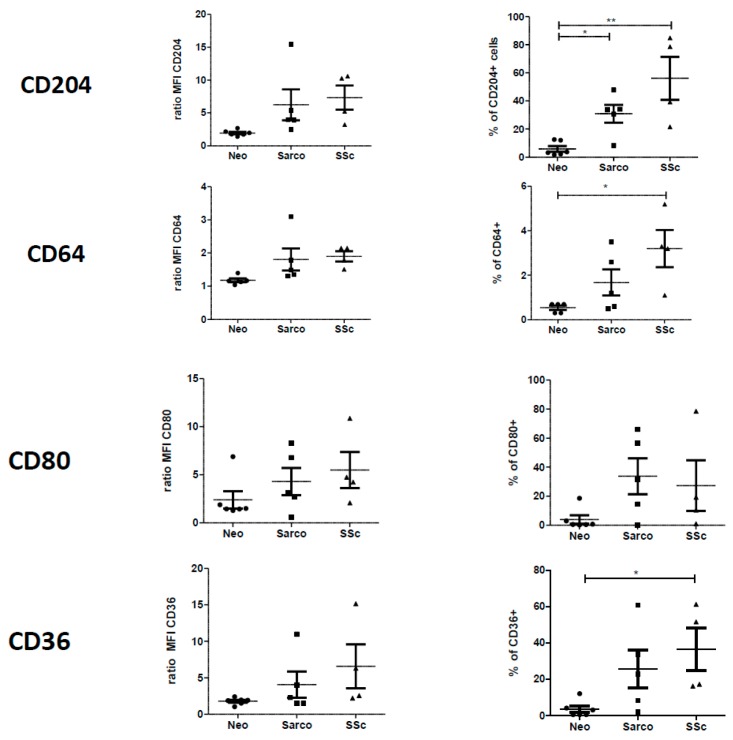
Comparison of cell surface molecules expression of alveolar MΦ (AM) from patients suffering of lung neoplasia (Neo), sarcoidosis (Sarco) or SSc-ILD. Bronchoalveolar lavages fluids of patients were washed and cells were plated until the following day. Cells were then harvested, stained and the expression of cell surface molecules was analyzed by flow cytometry. Data are expressed as mean fluorescence intensity (MFI) relative to isotype control (ratio) +SEM (**A**) and as percentage of positive cells +SEM (**B**) for five or six lung neoplasia, five sarcoidosis and four ILD-SSc. ANOVA followed by Newman–Keuls’ multiple comparison Test, * *p* < 0.05 and ** *p* < 0.01.

We next compared the expression of these markers on AM obtained from BAL of patients suffering from the three lung diseases of interest taken separately: (a) lung cancers (Neo group), (b) sarcoidosis (Sarco group) or (c) SSc-ILD (SSc group). Flow cytometry graphs are shown in supplementary Figure S2. The expressions of CD206 and CD169 were significantly higher in SSc-ILD patients when compared to Neo patients (Figure 2). By contrast, the expressions of the other markers were similar in Neo, Sarco and SSc-ILD patients. However, a significant increase in the percentage of positive cells was observed in SSc-ILD group for CD163, CD169, CD204, CD64 and CD36 when compared to Neo group and for CD163 and CD169 when compared to Sarco group (Figure 2). Moreover, the percentage of positive cells for CD204 was also significantly higher in AM from Sarco group in comparison with AM from Neo group (Figure 2).

### 2.3. Co-Expression of CD206, CD163 and CD169 in Blood-MDMs and AM

**Figure 3:**
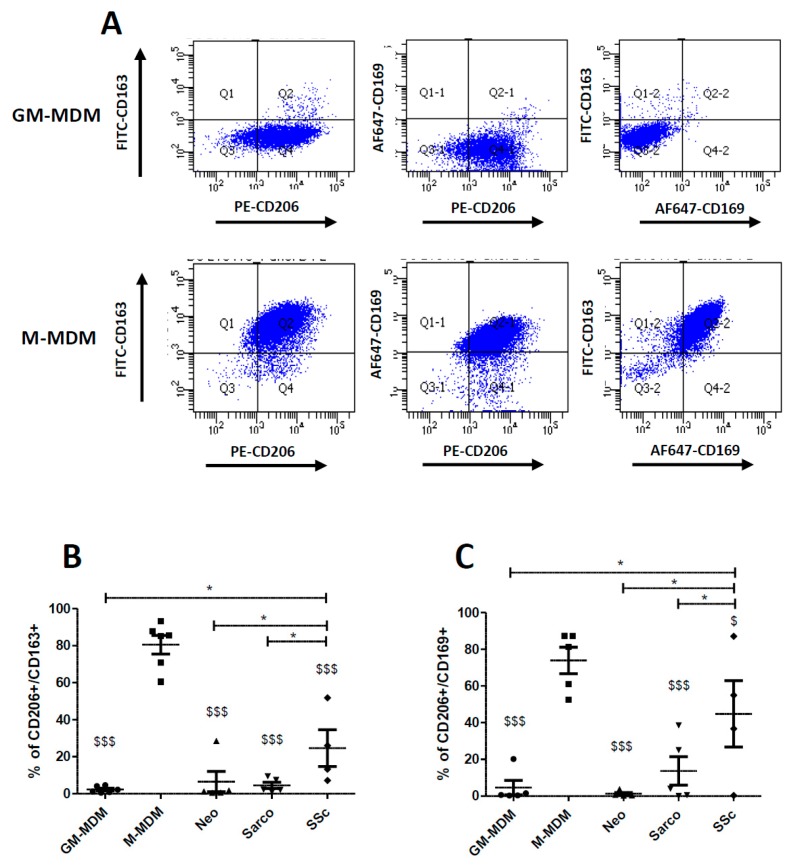
Comparison of the percentage of cells co-expressing CD206, CD163 and CD169 between GM-CSF and M-CSF-derived MDMs and AM from patients suffering of lung neoplasia, sarcoidosis or SSc-ILD. Primary human monocytes from healthy donors were differentiated into MDMs in vitro in the presence of GM-CSF (GM-MDM) or M-CSF (M-MDM) for 6 days. Bronchoalveolar lavage fluids of patients were washed and cells were plated until the following day. Cells were then harvested, stained and the expression of cell surfaces molecules was analyzed by flow cytometry. Graphs representing the percentage of GM-CSF or M-CSF-derived MDMs co-expressing CD206/CD163, CD206/CD169 or CD163/CD169 are representative of 5 independent experiments (**A**). Data expressed as the percentage of CD206+/CD163+ cells +SEM (**B**) or of CD206+/CD169+ cells +SEM (**C**) are the means of 5 independent experiments except for SSc-ILD with 4 samples. ANOVA followed by Newman–Keuls’ multiple comparison Test: * *p* < 0.05; $ *p* < 0.05 and $$$ *p* < 0.001 when compared to M-MDM.

Because, the expression and/or the percentage of positive CD163 and CD169 cells were increased in SSc-ILD and considering that CD206 expression seemed lower in AM from Neo group in comparison with those from Sarco or SSc groups (Figure 2), we analyzed their co-expression with CD206, a marker well expressed both by GM-MDMs and M-MDMs. The Figure 3A confirmed the data from Figure 1 showing that GM-MDMs were CD163^neg^ and CD169^neg^. By contrast, M-MDM CD206+ cells co-expressed both CD163 and CD169 (Figure 3).

The percentages of CD206+/CD163+ cells and of CD206+/CD169+ cells were significantly higher in SSc-ILD than Neo, Sarco and GM-MDMs, confirming the phenotypic difference of AM from this fibrotic lung disease (Figure 3B,C). Even if the percentages of CD206+/CD163+ cells and of CD206+/CD169+ cells of SSc-ILD were closer to M-MDMs, a significant difference remained between these two types of macrophages (Figure 3B,C).

### 2.4. Phenotypic Variations of Blood-MDMs from Healthy Donors and SSc Patients, and Comparisons with AM in SSc-Associated Lung Disease

**Figure 4:**
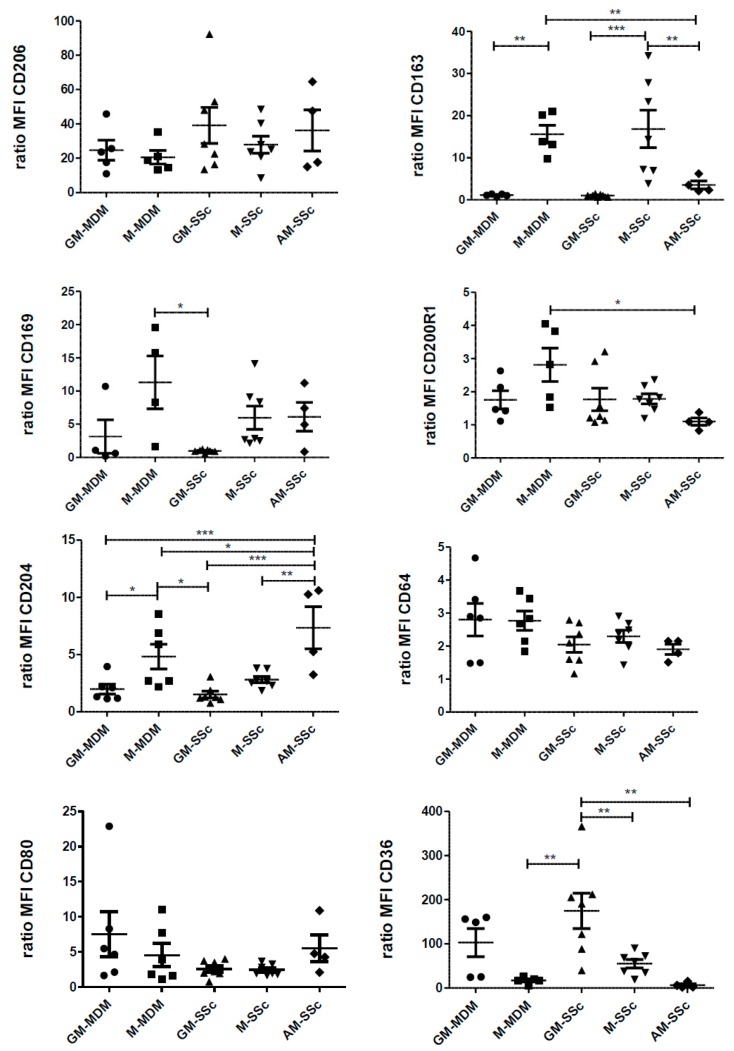
Comparison of cell surface molecule expression of GM-CSF and M-CSF-derived MDMs from healthy donors and from SSc patients with alveolar MΦ (AM) from patients with SSc-ILD. Primary human monocytes from healthy donors or SSc patients were differentiated into MDMs in vitro in the presence of GM-CSF (GM-MDM or GM-SSc) or M-CSF (M-MDM or M-SSc) for 6 days. Culture media were replaced between the 6th–7th day. Bronchoalveolar lavage fluids were washed and cells were plated until the following day. Cells were harvested, stained and the expression of cell surface molecules was analyzed by flow cytometry. Data are expressed as mean fluorescence intensity (MFI) relative to isotype control (ratio) +SEM for at least five healthy donors, or 7 SSc patients and 4 AM from patients with SSc-ILD. ANOVA followed by Newman–Keuls’ multiple comparison Test, * *p* < 0.05; ** *p* < 0.01 and *** *p* < 0.001.

Because BAL fluids from SSc were rare and that cell quantity was poor, we characterized MDMs from patients suffering of SSc, which were differentiated in the presence of GM-CSF (GM-SSc) or of M-CSF (M-SSc). The Figure 4 shows the compared expressions of the 8 macrophagic markers in healthy MDMs, SSc MDMs differentiated by GM-CSF or M-CSF and AM from SSc-ILD. The expression of CD206, CD64 and CD80 was similar in AM-SSc and in other macrophages (Figure 4). On the contrary, the expression of CD200R1 in AM-SSc was only found significantly lower than M-MDMs from healthy donors. The expression of CD163 was significantly lower in AM-SSc than in healthy or SSc macrophages differentiated by M-CSF but was similar to those of macrophages differentiated by GM-CSF (Figure 4). By contrast, the expression of CD204 was significantly higher in AM-SSc than in healthy or SSc macrophages differentiated by GM-CSF and also higher than in M-SSc. The expression of CD36 was similar in AM-SSc and in healthy or SSc macrophages differentiated by M-CSF and was significantly lower than in GM-SSc. The expression of CD169 also tended to be similar in M-SSc and AM-SSc in comparison with GM-SSc. Globally, the differences concerning the expression of surface markers between AM-SSc and the other macrophages mainly concerned scavenger receptors (CD163, CD204, CD36). Therefore, especially considering the expressions of CD204 and CD36, AM-SSc tended to be phenotypically closer to MDMs differentiated by M-CSF rather than GM-CSF. Beyond scavenger receptors, the expression of CD169 in AM-SSc in comparison with GM-SSc also tended to support these phenotypic considerations.

### 2.5. Secretion Levels of the Pro-Inflammatory Cytokine IL-6 and the Pro-Fibrotic Chemokine CCL18

**Figure 5:**
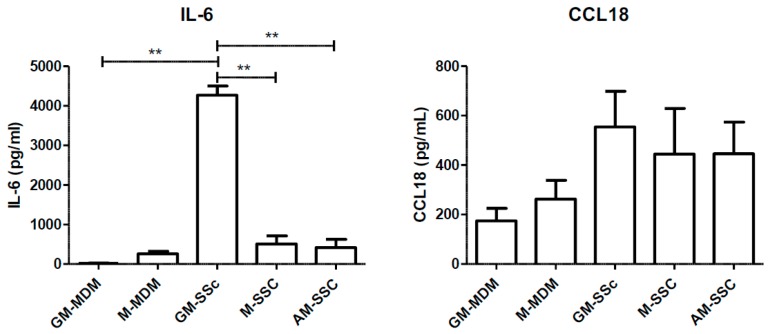
Comparison of IL-6 and CCL18 secretion levels in GM-CSF and M-CSF-derived MDMs from healthy donors and from SSc patients, with alveolar MΦ (AM) from patients with SSc-ILD. Primary human monocytes from healthy donors or SSc patients were differentiated into MDMs in vitro in the presence of GM-CSF (GM-MDM or GM-SSc) or M-CSF (M-MDM or M-SSc) for 6 days. Culture media were replaced at day 6 and, 24 h later, conditioned media were removed, stocked and ELISA were performed. Data are expressed as the mean of concentration in pg/mL + SEM from at least 5 healthy donors, or 7 SSc patients and four AM from patients with SSc-ILD. ANOVA followed by Newman–Keuls’ multiple comparison Test, ** *p* < 0.01.

In order to support our data on the phenotype in SSc-ILD, we quantified the secretion levels of IL-6, a pro-inflammatory cytokine, and CCL18, a chemokine found in pro-fibrotic lung tissue. IL-6 secretion was significantly higher in GM-SSc than M-SSc and than GM-MDMs. By contrast, IL-6 levels of AM-SSc were similar to those of M-MDM or M-SSc (Figure 5). There were no statistical differences in CCL18 secretion. Therefore, M-SSc and M-MDMs were not different from AM of SSc-ILD.

## 3. Discussion

Pulmonary macrophages are poorly available in human to perform in vitro studies due to a limited access to broncho-alveolar lavage (BAL) and to an even more limited access to IM. This statement is especially true considering rare systemic inflammatory and fibrotic lung disorders in which macrophages could play a key pathogenic role [6,7,9]. Moreover, phenotypic and functional differences between murine and human lung macrophages and monocytes exist, limiting conclusions drawn from mice models [1]. Relevant in vitro human macrophage models are, therefore, needed and blood MDM is an interesting and widely accepted approach [11]. As patients with anti-GM-CSF antibodies or with genetic defects on the GM-CSF pathway like GATA-2 deficiency, present severe lung involvement such as pulmonary alveolar proteinosis [15,16], the GM-CSF MDM is generally considered the most relevant model to simulate human AM in vitro. Based on two flow cytometry panels, exploring 8 key macrophage polarization markers in human, our data are consistent with this statement when considering AM globally with no distinction made between each specific lung disease.

We did not obtain BAL samples from healthy subjects in this study, however, AM of patients suffering from lung cancers were obtained from the alveolar lumen and were thus not specifically in contact or inside the tumor tissues, as the collection of macrophages inside the tumor would imply obtaining direct histopathological samples of the cancer. Therefore, AM from these patients could be considered closer to healthy AM rather than authentic tumor associated macrophages (TAMs).

Recent insights concerning macrophages in inflammatory and fibrotic conditions have shed light on the necessity of considering macrophage polarization in a given micro-environment, influenced both by the considered organ but also by the disorder affecting this organ [13,17]. Martinez et al. called for a dynamic view of the polarization process “to take into account the multiple elements in their systemic and local milieu and to define the kinetics, plasticity, reversibility, and memory of their responses in order to encompass the full functional range of activated macrophages” [13]. The polarization state of macrophages in various lung diseases may therefore vary and the most relevant MDM model could depend on the disease at stake. Our results demonstrate that the GM-CSF MDM model may fail to properly and fully reflect lung macrophage polarization states and phenotypes in fibrotic disorders such as systemic sclerosis [18,19,20]. This question is all the more important since macrophages could constitute relevant therapeutic targets to treat the deadly pulmonary manifestations of this inflammatory and fibrotic disorder [21,22].

In our work, the proportion of AM expressing CD204 of patients suffering from SSc-ILD is significantly enhanced, in comparison with AM of patients suffering from lung cancer. The expression of CD204 (scavenger receptor I Class A), in AM from SSc-ILD and M-CSF MDMs is an important feature since CD204 seems to play an important role in the pathogenesis of lung fibrosis [23]. CD204, is a homotrimeric glycoprotein composed of three 77-kDa monomers and is present on tissue macrophages of the spleen, thymus, heart, gut, liver and lungs [24,25,26]. CD204 null mice fail to develop fibrosis following silica exposure, in a mice model of chronic silicosis with pulmonary fibrosis characterized by typical focal lesions, interstitial thickening with increased connective tissue matrix, and cellular infiltrate into air space [24]. CD204 may play a role in shifting macrophages from an M1 pro-inflammatory state to an M2 state initiating the resolution phase but also fibrosis in case of uncontrolled M2 activation [24]. In human, CD204 is also involved in the pathogenesis of pulmonary fibrosis. Expression of CD204 is enhanced in AM of patients suffering from idiopathic pulmonary fibrosis (IPF), a chronic ILD sharing common features with SSc-ILD. This overexpression of CD204 in IPF, promotes the secretion of profibrotic markers such as CCL18, through a process involving the recognition of collagen type I by AM in a CD204 dependent manner [27]. Moreover, in our work, the percentage of cells expressing CD204 was also higher in patients with sarcoidosis ILD in comparison with patients suffering from lung cancers. As the pathophysiology of sarcoidosis ILD also includes fibrotic processes, this result is another evidence supporting the involvement of CD204 in the pathogenesis of lung fibrosis [28,29]. There is nonetheless no clear evidence for an imbalanced macrophage polarization in sarcoidosis and our results are consistent with this data [30]. However, previous studies have reported that AM from sarcoidosis patients produce higher amount of the profibrotic cytokine CCL18, a mediator also overexpressed in IPF and SSc [6,30,31,32].

In our study, AM of patients suffering from SSc-ILD, in comparison with AM from lung cancers, also had a significantly enhanced expression of the M2 polarization marker CD206 and a higher percentage of positive cells for CD169 and CD163. In the literature, skin macrophages of SSc patients with skin fibrosis also have a higher expression of CD169 and CD163 [9,20,28]. Circulating monocytes of SSc patients also present enhanced expressions of CD163, CD169 and CD206 [17,33,34] Therefore, macrophages from various tissues in SSc show similar phenotypes and polarization signatures [33]. In our study, the expression of CD169 and CD163 was also significantly higher in M-CSF MDMs in comparison with GM-CSF MDMs, stressing the relevance of considering M-CSF MDMs as the most adapted in vitro macrophage model for SSc. In a macaque model of ILD, Cai et al. have demonstrated that CD163 positive monocytes are precursors of IM that secondly become recruited AM in pathological conditions [35]. These new recruited AM co-express CD163 and CD206 [35]. Considering these results and as suggested by Martinez et al. [13], we pointed the need to focus not only on macrophage at a population level but also at a single-cell level. This is why we studied macrophages co-expressing CD206/CD163 and CD206/CD169. Our results demonstrated that the percentages of CD206+/CD163+ cells were significantly higher in SSc-ILD than in lung cancer or sarcoidosis and/or GM-MDMs. Considering results from Cai et al. [35], this phenotype is concordant with the one of recruited AM. We can hypothesize that these CD206/CD163 positive AM in SSc might be recruited AM, derived from IM or blood monocytes. Consistently, these CD206/CD163 and CD206/CD169 positive populations were highly represented in M-CSF MDMs whereas they were almost absent in GM-MDMs. These results on co-expressed markers also support the M-CSF MDM model as a relevant alternative to simulate AM of fibrotic lung disorders like SSc. Recent insights in the pathogenesis of SSc have also underscored the importance of STAT3 as a key integrator of profibrotic signaling [36]. M-CSF can induce the expression of CD163 in MDMs in a STAT3-dependent manner, whereas GM-CSF MDMs do not express CD163, once again supporting the relevance of the M-CSF MDM model [13,35,36,37,38]. In a mouse model of bleomycin-induced lung fibrosis, M-CSF null mice had a marked reduction in the number of AM and in pulmonary fibrosis features when compared with M-CSF wild type mice, supporting once again the importance of M-CSF in the pathogenesis of fibrotic lung diseases and the relevance of M-CSF MDMs to explore these disorders. One of the limits of our study is the lack of information concerning IM in our patients, since only AM from BAL were collected as we did not study or have access to any lung biopsy. Therefore, the hypotheses concerning the ontogeny of lung macrophages in ILD as suggested above must be considered carefully and further studies are warranted to answer these questions. Another limitation is the small sample size due to the rarity of the diseases of interest. Indeed the BAL recovery of SSc-ILD was rare and, in each sample, the number of AM was poor, and we were therefore unable to perform both flow cytometry panels on 2 samples, limiting the number of patients included in each analysis. Nonetheless this limitation highlights the need for relevant blood MDM models to study the pathogenesis of these complex and rare systemic disorders.

To support these phenotypic considerations on AM in SSc-ILD, we compared the secretion of the pro-inflammatory cytokine IL-6 and the pro-fibrotic chemokine CCL18 by MDMs and SSc-AM. We have chosen these secreted markers as they are both respectively considered highly representative of the inflammatory and fibrotic processes involved in the pathogenesis of SSc [31,32,39,40]. Considering IL-6, GM-MDMs from SSc patients had a significantly increased secretion of this cytokine in comparison with MDMs from healthy donors but also in comparison with M-MDMs and AM from SSc patients. IL-6 levels from AM in our study are concordant with previous results on BAL fluid in SSc-ILD [41]. Our results illustrate two well-known concepts: 1/ in comparison with M-CSF, GM-CSF promotes the secretions of IL-6 by MDMs in inflammatory conditions [42] and 2/IL-6 expression is globally increased in SSc MDMs [39,40]. Nonetheless, the IL-6 levels of AM from SSc-ILD were more similar to M-SSc rather than GM-SSc. Concerning CCL18, there were no major differences between all types of MDMs and AM. Considering the combined results on secretion levels of IL-6 and CCL18, SSc-AM were therefore more similar to M-SSc rather than GM-SSc, supporting the results from the phenotypic evaluation. A wider and more refined assessment is nonetheless warranted, to confirm these first results on comparative functional evaluations of MDMs and AM.

By illustrating that M-CSF MDMs share some phenotypic features with human lung AM from fibrotic disorders such as SSc, our work reinforces the vision of a wide and heterogeneous functional range of activated macrophages, not only depending on the organ of interest but also on the pathological disorder at stake [1,2,13,33]. In this pathogenic perspective, our study was focused on phenotypic considerations based on two flow cytometry panels, exploring 8 key macrophage polarization markers in human and highlighting the relevance of each MDM model depending on the disease of interest. Two key secreted markers of SSc (IL-6 and CCL18) were also evaluated, showing concordant results with the phenotypic evaluation. However, the next step in exploring M-CSF and GM-CSF blood MDMs and their connections with AM from various disorders in human, will be a more precise assessment of their functional properties in term of secretion of pro-inflammatory, pro-fibrotic and pro-angiogenic cytokines or markers (beyond IL-6 and CCL18), in comparison with AM. This wider functional approach may help in identifying new therapeutic targets in the field of rare lung diseases such as fibrotic or inflammatory ILDs.

## 4. Materials and Methods

### 4.1. Isolation and Culture of Monocyte Derived Macrophages

Peripheral blood mononuclear cells were obtained from blood buffy coats from healthy donors (provided by Etablissement Français du Sang, Rennes, France) through Ficoll gradient centrifugation. Monocytes, selected after 1-h adhesion step, were differentiated into macrophages for 6 days using GM-CSF (400 IU/mL) (also known as sargramostim, Sanofi-Avantis-Montrouge, Paris, France) or M-CSF (50 ng/mL) from Miltenyi Biotec SAS (Paris, France) in RPMI 1640 medium GlutaMAX (Gibco, Life technologies SAS, Courtaboeuf, France) supplemented with 10% (*v*/*v*) of heat-inactivated fetal bovine serum (FBS, Lonza, Levallois-Perret, France) and antibiotics. At day 6, culture media were replaced with a fresh medium with 5% of FBS. At day 7, conditioned media were removed and stocked at −20 °C for ELISA analysis whereas the cells were washed and harvested for flow cytometry analysis.

### 4.2. Characteristics of Patients

The experiments were conducted according to the International Conference on Harmonization (ICH) Good Clinical Practice (GCP) guideline (1997) and approved by the Ethic Committees of Rennes University Hospital (Caract-Aires Project, N°16.122) and by the “Commission nationale de l’informatique et des libertés (CNIL)” N° 1998854 (17 October 2016). Patients were recruited by the Pulmonology department of Rennes University Hospital and all of them gave written informed consent. The clinical features of patients are reported in Table 1.

### 4.3. Bronchoalveolar Lavages (BALs)

BALs were performed under local anesthesia with 2% lidocaine. BALs were obtained from 6 patients suffering from lung cancers (Neo), from 5 sarcoidosis (Sarco) patients and from 5 patients with systemic sclerosis (SSc). BAL collecting was performed in accordance with the standard procedures using a fiberoptic bronchoscope. About 100 mL of sterile 0.9% saline was instilled in the bronchial and alveolar lumen, and then, aliquoted and recovered into sterile propylene tubes. The average volume obtained for this study by our lab was about 10 mL. BALs were then centrifugated at 450 rcf for 5 min, washed once with 5 mL of PBS 1× and suspended in RPMI 1640 Glutamax containing 10% FCS and antibiotics and plated at 0.14 × 10^6^ cells/cm^2^. After an adhesion step of 3 h, the medium was replaced with 5% of FBS. The next day, conditioned media were removed and stocked at −20 °C for ELISA analysis whereas the cells were washed and harvested for flow cytometry analysis.

### 4.4. Flow Cytometry Analysis

Phenotypic analysis of monocytes-derived macrophages and of macrophages from BALs was performed using flow cytometric direct immunofluorescence. Cell detachment was performed using Accutase^TM^ (Biolegends, London, UK), in order to maintain expression of M2 membrane marker [43]. A first step of viable cell staining was performed using Fixable Viability Stain 450 (BD Biosciences, San Jose, CA, USA) for 10 min at room temperature. After a washing step, cells were then blocked in phosphate-buffered saline (PBS) supplemented with 2% FCS solution and with Fc block (Miltenyi Biotec SAS, Paris, France) for 10 min at room temperature to avoid nonspecific binding, and then re-suspended and incubated with specific Ab or appropriate isotypic controls for 30 min at 4 °C. Cells were then washed once with PBS and analyzed using LSR II cytometer and FACSDiva software (BD Biosciences). The phenotype characterization of macrophages was performed using two panels. The antibodies used for panel 1 were: FITC anti-CD64, PE anti-CD204 (SRA1), APC anti-CD200R1 and PeCya7 anti-CD80 (all from BD Biosciences). The antibodies used for panel 2 were: FITC anti-CD163, PE anti-CD206, AlexaFluor 647 anti-CD169 (BD Biosciences) and PeVio770 anti-CD36 (Miltenyi). Results were expressed as the percentage of positive cells or as the ratio of mean (of median) of fluorescence intensity (MFI) calculated as follows: mean fluorescence (mAb of interest)/mean fluorescence (isotype control mAb).

### 4.5. Quantification of Cytokine and Chemokine Levels

Levels of IL-6 and CCL18 secreted in culture media were quantified by ELISA using specific Duoset ELISA development system kits (R&D Systems, Abingdon, UK).

### 4.6. Statistical Analysis

Data were analyzed using GraphPad prism software (GraphPad Software, La Jolla, CA, USA). Student’s *t*-test or One-way analysis of variance (ANOVA) followed by Newman–Keuls’ post-hoc test were applied for statistical analysis and *p*-values < 0.05 were considered significant.

## Figures and Tables

**Table 1:** Clinical characteristic of patients.

Patient’s Characteristics	ILD-SSc	Sarcoidosis	Neoplasia	All
*n*	5	5	6	16
Age + SD (in year)	62.6 + 8.5	54.2 + 7.4	55.2 + 9.9	57.2 + 9.1
number of women (% of women)	3 (60)	0 (0)	2 (33)	5 (31)
Number of Current smoker or ex-smoker (in %)	2 (40)	2 (40)	6 (100)	10 (67)

## Supplementary Materials

Supplementary materials can be found at http://www.mdpi.com/1422-0067/19/3/894/s1.
