# Supplementary material for: Distinct Properties of Human M-CSF and GM-CSF Monocyte-Derived Macrophages to Simulate Pathological Lung Conditions In Vitro: Application to Systemic and Inflammatory Disorders with Pulmonary Involvement
Source: Int J Mol Sci. 2018 Mar 17;19(3):894. doi: 10.3390/ijms19030894 (PMC5877755; doi:10.3390/ijms19030894)
Supplement: Supplementary file 1 [file ijms-19-00894-s001.pdf]

Supplementary Figure 1

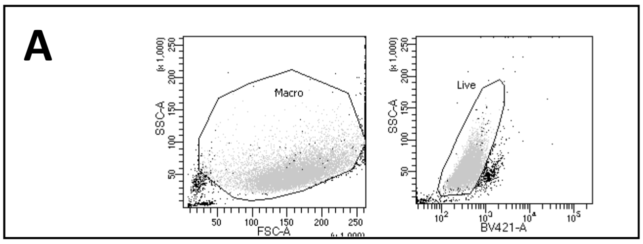

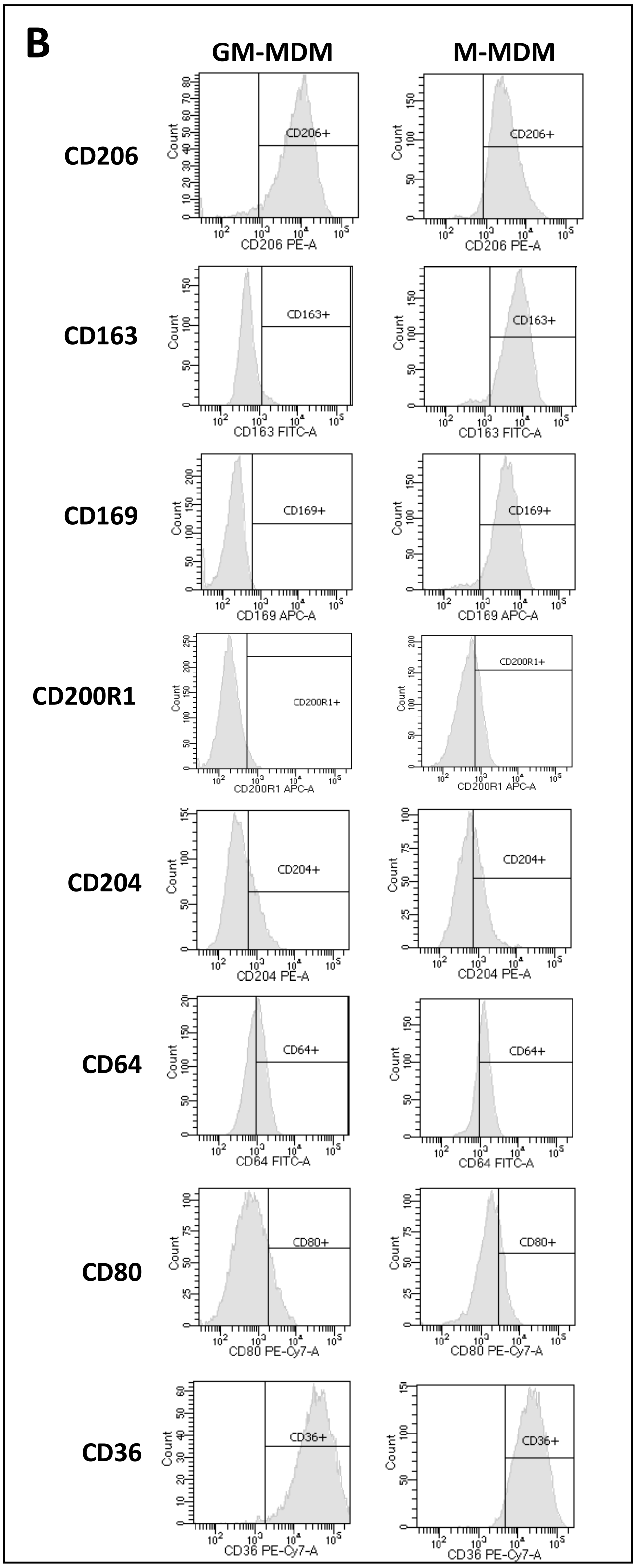

Supplementary Figure 2

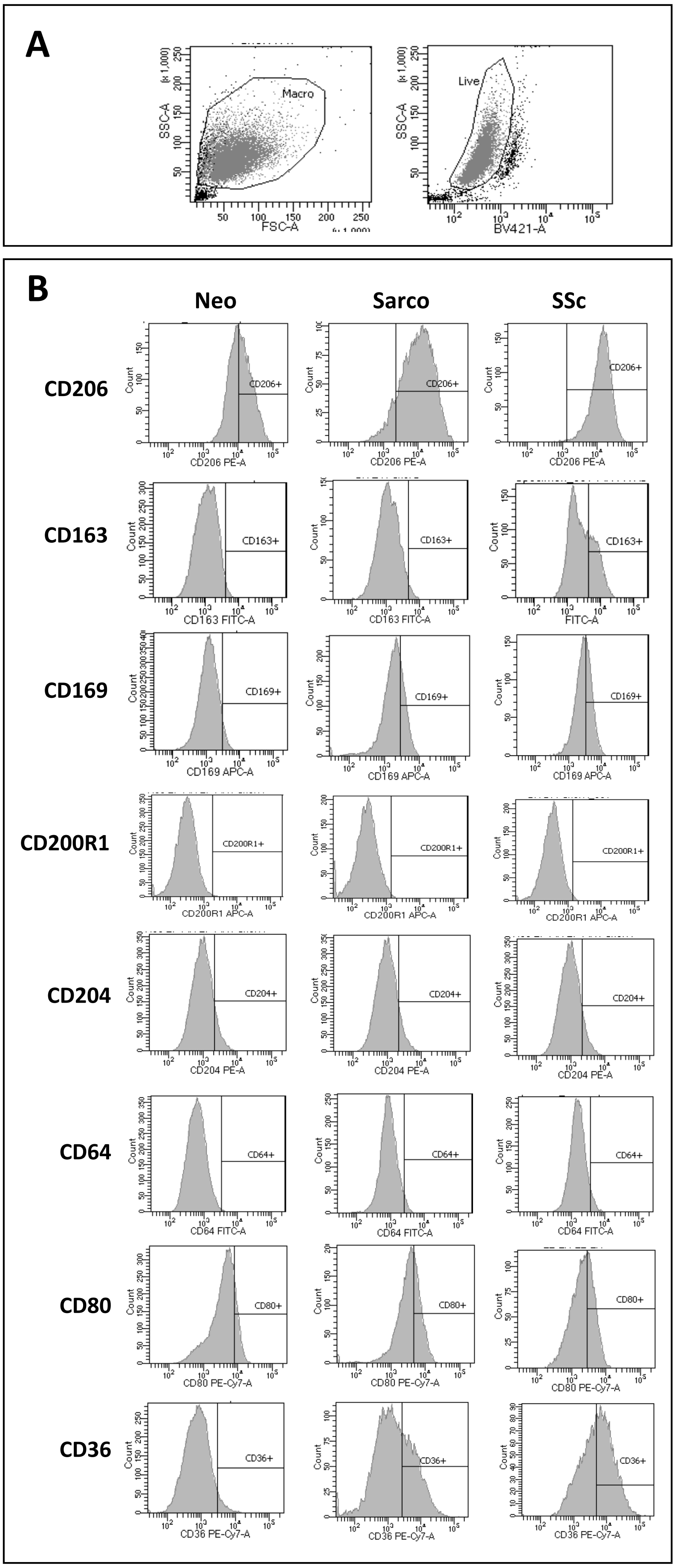

Legends of supplementary data

Supplementary Figure 1: Phenotype differences between GM-CSF and M-CSF MDMs from healthy donors. (A) Gating strategy to exclude dead cells, stained with Fixable Viability Stain 450. (B) GM-MDM and M-MDM were stained with antibodies for flow cytometry analysis. The negative threshold was placed according to MDMs stained with isotype control antibodies. Histograms are representative of independent cultures of MDMs from 6 healthy donors.

Supplementary Figure 2: Phenotype difference between alveolar MΦ (AM) of patients suffering from lung neoplasia (Neo), sarcoidosis (Sarco) or SSc-ILD. (A). Gating strategy to exclude dead cells, stained with Fixable Viability Stain 450. (B) AM were stained with antibodies for flow cytometry analysis. The negative threshold was placed according to AM stained with isotype control antibodies. Histograms are representative of at least 4 independent cultures of AM.
